# Noncompaction Cardiomyopathy with Charcot-Marie-Tooth Disease

**DOI:** 10.1155/2015/646890

**Published:** 2015-06-09

**Authors:** Sherif Ali Eltawansy, Andrea Bakos, John Checton

**Affiliations:** ^1^Internal Medicine Department, Monmouth Medical Center, Long Branch, NJ 07740, USA; ^2^Drexel University College of Medicine, Philadelphia, PA 19129, USA; ^3^Cardiology Department, Monmouth Medical Center, Long Branch, NJ 07740, USA

## Abstract

We report a case of a 53-year-old female presenting with a new-onset heart failure that was contributed secondary to noncompaction cardiomyopathy. The diagnosis was made by echocardiogram and confirmed by cardiac MRI. Noncompaction cardiomyopathy (also known as ventricular hypertrabeculation) is a newly discovered disease. It is considered to be congenital (genetic) cardiomyopathy. It is usually associated with genetic disorders and that could explain the genetic pathogenesis of the non-compaction cardiomyopathy. Our case had a history of Charcot-Marie-Tooth disease. There is a high incidence of arrhythmia and embolic complications. The treatment usually consists of the medical management, defibrillator placement, and lifelong anticoagulation. Heart transplantation will be the last resort.

## 1. Case Presentation

**Figure 1:**
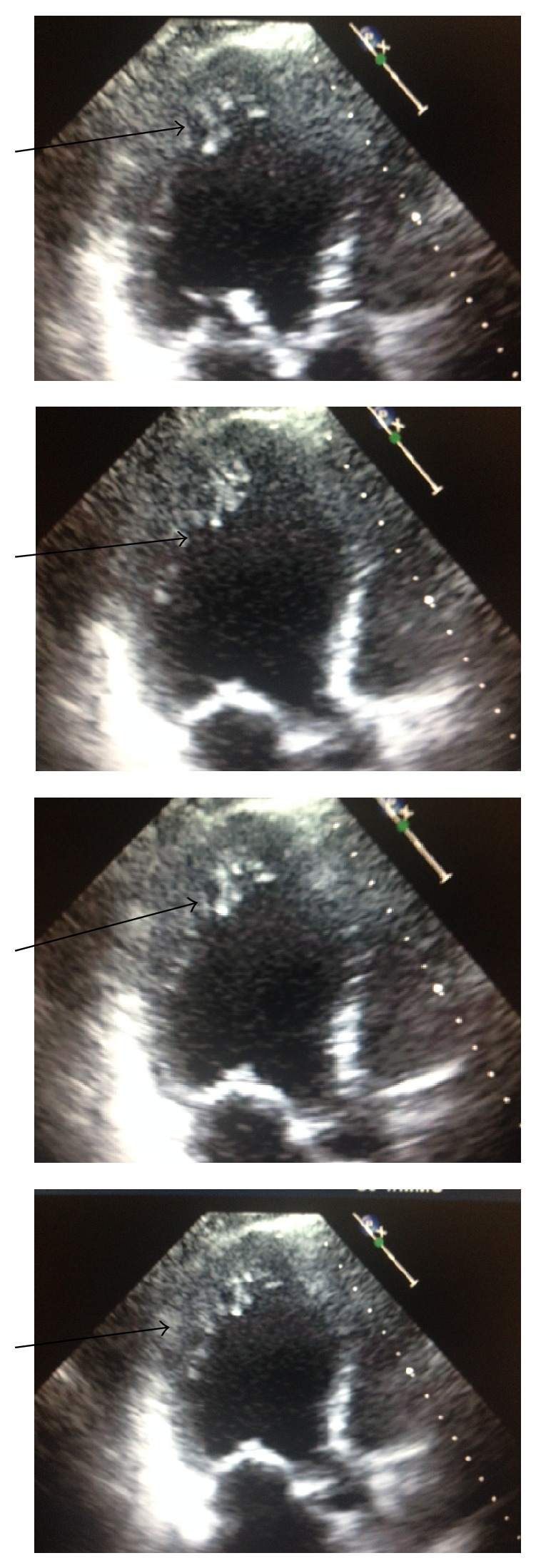
Echocardiogram.

We report a case of a 53-year-old Caucasian female who started to experience shortness of breath on exertion few months before presentation to us. She was admitted to a hospital in another state as she was on a trip due to worsening shortness of breathing. She was initially diagnosed as a case of congestive heart failure and was given furosemide and she improved. According to her medical records, she had a CT scan of the chest with angiography to exclude pulmonary embolism and it was negative. She was not known for any congenital heart disease, diabetes mellitus, or hypertension. She had a history of Charcot-Marie-Tooth disease with neuropathy. Her surgical history included uterine fibroid embolization 10 years ago and history of cat scratch disease 20 years ago. She went back home and was still taking oral furosemide. She was single and was working in a software company. She denied history of smoking, alcohol, or drug dependence. Review of system was negative apart from exertional dyspnea, orthopnea, and nocturnal dyspnea. Chronic foot pain with weakness and high arched foot was secondary to the history of Charcot-Marie-Tooth disease. By examination, body mass index was 29.84 kg/m^2^. By auscultation, there was a holosystolic murmur grade 2/6 in the lower left sternal border. There were no rales, rhonchi, or wheezes on auscultation at the time of presentation to us. The patient was then referred by her primary physician to the cardiologist office. EKG was done and showed sinus bradycardia with heart rate of 54/minute. There were nonspecific ST-T wave changes. Patient had an echocardiogram (Figure 1) showing left ventricle moderately dilated with severely reduced systolic function.

Ejection fraction was 25%. There was severe hypokinesis of the anteroseptal region with mild hypokinesia of the inferolateral wall. Right ventricle systolic function was moderately to severely reduced. There were severe mitral regurgitation, tricuspid regurgitation, dilated inferior vena cava, and increased right atrial pressure. Patient did a nuclear stress test showing abnormal myocardial perfusion of the left ventricle, left ventricular dilation, and area of thickening of the anterolateral portion of the left ventricle. It also showed severe global hypokinesia of the anterior wall and interventricular septum. The clinical, EKG, and hemodynamic response was normal. Patient was started by the cardiologist on carvedilol, lisinopril, vitamin D2, and warfarin for anticoagulation in addition to the furosemide. Laboratory work included the following. Hemoglobin was 109 g/L, WBC was 8.8 × 10^9^/L, platelets were 239 × 10^9^/L, prothrombin time was 12.9 seconds, INR was 1.2, partial thromboplastin time (PTT) was 30.5 sec, serum creatinine was 61.83 *µ*mol/L, blood urea nitrogen was 7.84 mmol urea /L, serum sodium [Na^+^] was 143 mmol/L, and serum potassium [K^+^] was 4.3 mmol/L. Cardiac enzymes: troponin I baseline was 0.02 *μ*g/L. B-natriuretic peptide was 194 pg/mL.

Patient was scheduled for elective cardiac catheterization. The catheterization showed the following. Hemodynamic assessment demonstrated mild systemic hypertension, moderately to severely elevated left ventricular end-diastolic pressure, severely depressed cardiac output, markedly elevated pulmonary capillary wedge pressure, mildly to moderately elevated systemic vascular resistance, and moderately elevated pulmonary vascular resistance. There were severe left sided failure and moderate to severe right sided failure. There was moderate pulmonary hypertension. There was no angiographic evidence for occlusive coronary artery disease. Global left ventricular function was severely depressed. EF calculated by contrast ventriculography was 22%, EF by echo was 25%, and EF by radionuclide angiography was 29%. The left ventricle was moderately dilated. There was no mural thrombus. The mitral valve exhibited severe regurgitation. Impression: the coronary anatomy is normal. Left ventricular function is markedly abnormal (noncompaction cardiomyopathy (NCC)). The patient had a Holter monitor showing infrequent premature ventricular contractions.

**Figure 2:**
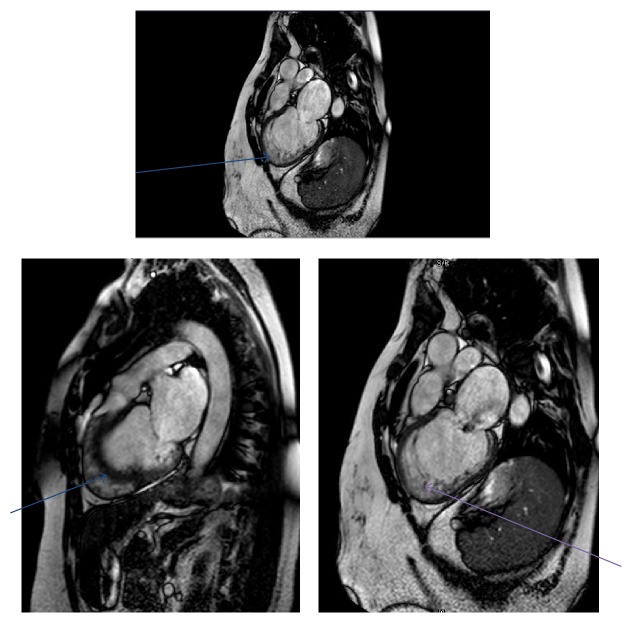
Cine MRI, morphology, phase-contrast, and contrast cardiac MRI.

Cine MRI, morphology, phase-contrast, and contrast cardiac MRI were performed (Figure 2).

Left ventricle: LVED volume is 262 mL, LVES volume is 208 mL, and LVEF is 20%. There were severe LV dilatation with normal LV wall thickness, severe diffuse LV hypokinesis, no regional akinesis or dyskinesis, severe LV dilatation with normal LV wall thickness, and severe diffuse left ventricular hypokinesis. No regional akinesis or dyskinesis was found. Contrast late enhancement study showed no evidence of segmental or significant patchy late contrast enhancement of myocardial wall, no evidence of infarction or infiltration, and no evidence of large area of myocardial edema or myocarditis. Cardiac MRI findings showed Severe MR (mitral regurgitation) secondary to left ventricular dilatation, mitral annular dilatation, and left ventricular dysfunction. These findings were most consistent with dilated cardiomyopathy. After the revision with the cardiologist and given the previous echocardiographic results, it was found that the apex of LV is almost circumferentially heavily trabeculated with a ratio of trabeculated endocardial layer (noncompacted layer, 21 mm) to myocardial layer (compacted myocardium, 7 mm) of >3 though there is no involvement of apical septum; in the midlevel of LV the lateral wall and some part of inferior wall and anterolateral wall are heavily trabeculated with a ratio of noncompact (14 mm) to compact layer (6.5 mm) of >2; the basal segment of LV is mostly spared from trabeculation except lateral wall which is minimally/mildly trabeculated. The papillary muscles appear sponge-like with smaller muscle bundles. Left ventricle noncompaction is suspected with prominent lateral wall and most apical noncompact layer as well as sponge-like papillary muscle appearance. Left ventricle end-diastolic volume is 262 mL and left ventricle end-systolic volume is 208 mL.

The patient had a Medtronic single chamber AICD (automated implanted cardioverter defibrillator).

## 2. Discussion 

*Noncompaction cardiomyopathy* (*NCC*), also called* spongiform cardiomyopathy*, is a rare congenital cardiomyopathy that affects both children and adults [1]. It results from the failure of myocardial development during embryogenesis [2]. During early embryonic development, the myocardium is a loose network of interwoven fibers separated by deep recesses that link the myocardium with the left ventricular cavity. Gradual “compaction” of this spongy meshwork of fibers and intertrabecular recesses, or “sinusoids,” occurs between weeks 5 and 8 of embryonic life, proceeding from the epicardium to endocardium and from the base of the heart to the apex. The coronary circulation develops concurrently during this process, and the intertrabecular recesses are reduced to capillaries [3]. The normal process of trabeculation appears to involve secretion of neuregulin growth factors from the endocardium and may also involve angiogenesis factors, such as vascular endothelial growth factor and angiopoietin-1 [4]. NVM (noncompaction of ventricular myocardium) was first described in association with other congenital anomalies, such as obstruction of the right or left ventricular outflow tracts, complex cyanotic congenital heart disease, and coronary artery anomalies [4]. The left ventricle is uniformly affected, but biventricular noncompaction has been reported, with right ventricular noncompaction described in less than one-half of patients [4]. Noncompaction cardiomyopathy was first identified as an isolated condition in 1984 by Engberding and Benber. They reported a 33-year-old female presenting with exertional dyspnea and palpitations. Investigations concluded persistence of myocardial sinusoids (now termed noncompaction) [5]. Trabeculation of the ventricles is normal, as are prominent, discrete muscular bundles greater than 2 mm. In noncompaction, there are excessively prominent trabeculations. Chin et al. described echocardiographic method to distinguish noncompaction from normal trabeculation. They described a ratio of the distance from the trough and peak, of the trabeculations, to the epicardial surface. Noncompaction is diagnosed when the trabeculations are more than twice the thickness of the underlying ventricular wall [6]. Histologically, isolated noncompaction differs from noncompaction associated with other congenital heart diseases in that the deep intertrabecular recesses communicate with the left ventricular cavity in the former and with both the coronary circulation and the left ventricle in the latter [7]. Both familial and sporadic forms of noncompaction have been described. In the original report of INVM, which predominantly involved children, familial recurrence was seen in half of patients. Familial recurrence was seen in 18% in the largest reported adult population with INVM (isolated noncompaction of ventricular myocardium) [6, 7].

Bleyl et al. reported a family of 6 affected children with INVM and X-linked inheritance. In this family, genetic linkage localized INVM to a mutation in the G4.5 gene of the Xq28 chromosome region, where other myopathies with cardiac involvement have been localized, including Barth syndrome, Emery-Dreifuss muscular dystrophy, and myotubular myopathy [8]. The cardiac-specific gene CSX has been implicated in the development of some cases of INVM. Distal chromosome 5q deletion has been reported to cause loss of the gene [9].

In the initial case series of isolated noncompaction [6], the median age at diagnosis was 7 years. Subsequent case reports have described this finding in adults, including the elderly [7]. In the largest series of patients with INVM, the prevalence was 0.014% of patients referred to the echocardiography laboratory. The true prevalence is unclear [7]. Isolated noncompaction is currently categorized as an unclassified cardiomyopathy by the World Health Organization classification, but a growing body of literature on the characteristic features of INVM has led some to call for its designation as a distinct cardiomyopathy [7]. Three major clinical manifestations of noncompaction have been described: heart failure, arrhythmias, and embolic events [3]. Findings vary among patients, ranging from asymptomatic left ventricular dysfunction to severe, disabling congestive heart failure. Over two-thirds of the patients in the largest series with INVM had symptomatic heart failure [7]. Both systolic and diastolic ventricular dysfunctions have been described. Restrictive hemodynamics by cardiac catheterization as well as an initial presentation of INVM as a restrictive cardiomyopathy has been described in children with INVM [10]. Systolic dysfunction could be coming from subendocardial perfusion defect which has been described in INVM using cardiac magnetic resonance imaging (MRI) [11]. Positron emission tomography (PET) [12] and scintigraphy with thallium-201 [10] have demonstrated transmural perfusion defects correlating with areas of noncompacted myocardium in INVM. Junga et al. suggested that altered perfusion and coronary flow reserve in INVM may be related to failure of the coronary microcirculation to grow with the increasing ventricular mass, compression of the intramural coronary bed by the hypertrophied myocardium, or both processes [12]. Arrhythmias are common including atrial fibrillation, ventricular tachycardia, and sudden death [7, 13]. Embolic complications may be related to development of thrombi in the extensively trabeculated ventricle, depressed systolic function, or the development of atrial fibrillation [7]. An association between noncompaction and neuromuscular disorders has also been described, with as many as 82% of patients having some form of neuromuscular disorder [14]. Although echocardiography has been the diagnostic test of choice for noncompaction, other modalities have been used for the diagnosis, including contrast ventriculography, computed tomography [15], and MRI [12]. Standard medical therapy for systolic and diastolic ventricular dysfunction is warranted.

Cardiac transplantation has been used for those with refractory congestive heart failure. Only 6 cases of INVM leading to cardiac transplantation have been published to date [16]. Because of the frequency of ventricular tachycardia and significant risk of sudden cardiac death and systemic embolism, assessment for atrial and ventricular arrhythmias by ambulatory ECG monitoring should be performed annually. As more information is gathered about NVM and risk of sudden cardiac death, implantable defibrillator technology may have an expanded role [17]. Long-term prophylactic anticoagulation has been recommended [7]. Although the prognosis for patients with NVM varies, nearly 60% of patients described in one large series had either died or undergone cardiac transplantation within 6 years of diagnosis. Two of 8 in the initially asymptomatic group of this series died during the follow-up period, both having documented sustained ventricular tachycardia and one with sudden cardiac death [3].

Our reported case had a history of Charcot-Marie-Tooth disease which is related to the noncompaction cardiomyopathy discovered later. Whether there is a causal or pathogenic relation between LVHT (left ventricular hypertrabeculation) and Charcot-Marie-Tooth disease in the present patient remains to be established. Indications for a causal relation are as follows [17]. Having reviewed the literature, it appears that cardiac abnormalities are more frequent in Charcot-Marie-Tooth patients than in controls [18]. Myocardial involvement in a case like Charcot-Marie-Tooth disease could be due to partial homology of the PMP22 protein with other proteins expressed in the heart, such as EMP or MP20. LVHT, dilated cardiomyopathy, left bundle branch block, and heart failure may be associated with Charcot-Marie-Tooth hereditary neuropathy type 1A due to the PMP22 duplication on chromosome 17p11.2-12. A causal relation between the cardiac abnormalities and the mutation remains elusive. If a patient with a neuromuscular disorder is investigated echocardiographically, special attention should be directed towards the detection of LVHT [19]. In the majority of the cases, LVHT occurs in association with various genetic disorders [20]. Whether the relation between these conditions and LVHT is causal or coincidental is unknown. The frequent occurrence together with genetic disease, however, suggests that, though unproven, there is a pathogenetic link between the various mutations and the occurrence of LVHT. Genetic disorders associated with LVHT include cardiac disease other than LVHT, NMDs (neuromuscular disorders) with cardiac involvement, noncardiac, non-NMD hereditary disorders, and chromosomal aberrations [21].

## 3. Conclusion

Noncompaction cardiomyopathy (also known as ventricular hypertrabeculation) is a rare form of cardiomyopathy that is usually diagnosed by echocardiogram or cardiac MRI. Misdiagnosis and underreporting of the disease make it difficult to fully understand and study that disease, especially that it is a newly discovered and described entity of the cardiomyopathy. Correlation has been reported with other genetic diseases like Charcot-Marie-Tooth disease in our case.
